# The effect of narrative element incorporation on physical activity and game experience in active and sedentary virtual reality games

**DOI:** 10.1007/s10055-023-00754-7

**Published:** 2023-01-31

**Authors:** Amy Shirong Lu, Victoria Pelarski, Dar Alon, Aleksandra Baran, Emma McGarrity, Neha Swaminathan, Caio Victor Sousa

**Affiliations:** 1 Health Technology Lab, College of Arts, Media and Design, Bouvé College of Health Sciences, Northeastern University, Boston, MA 02115 USA; 2 Bloomberg School of Public Health, Johns Hopkins University, Baltimore, MD 21205 USA; 3 Harvard T.H. Chan School of Public Health, Harvard University, Boston, MA 02115 USA; 4 Health and Human Sciences, Frank R. Seaver College of Science and Engineering, Loyola Marymount University, Los Angeles, CA 90045 USA

**Keywords:** Virtual reality, Active video game, Exergame, Narrative, Story, Physical activity, Game experience

## Abstract

Narratives are pervasive in video games and have been found to increase physical activity in active video games. However, the effect of incorporating narrative elements has seldom been examined in fully immersive virtual reality games. We investigated the effect of narrative element incorporation (between-subject: narrative vs. no narrative) in active virtual reality and sedentary virtual reality games (within-subject) and examined between- and within-subject effects on physical activity behavior, game experience, and physical activity engagement. We randomized 36 sedentary college students to either the narrative or the non-narrative group. All participants played an active virtual reality and a sedentary virtual reality game in counter-balanced order. Before each game session, they either watched a 5-min narrative video (narrative) or directly played the original virtual reality games without narratives (non-narrative). We collected participants’ physical activity data using wrist-worn accelerometers; we obtained their game experience and physical activity engagement via questionnaires. The narrative group spent a greater proportion of time in moderate-to-vigorous physical activity (%) and had less non-movement time during the active virtual reality gameplay than the non-narrative group (all *p *values < .05). The active virtual reality sessions induced a greater positive affect and greater physical activity engagement ratings than the sedentary virtual reality sessions. The incorporation of narrative elements in active virtual reality increased the relative time spent in moderate-to-vigorous physical activity and reduced non-movement time, compared to the non-narrative group. Active virtual reality encouraged more activity by participants and offered them a more enjoyable gaming experience in which they engaged more. Active virtual reality is a feasible physical activity promotion option among sedentary adults; the incorporation of narrative elements in active virtual reality helps increase relative moderate-to-vigorous physical activity and should be further explored for its efficacy.

**Supplementary Information:**

The online version contains supplementary material available at 10.1007/s10055-023-00754-7.

## Introduction

Insufficient levels of physical activity are highly prevalent in US adults (U.S. Department of Health and Human Services 2018) and significantly increase the risk for diseases related to a sedentary lifestyle in later life (Lee et al. 2012). Active video games, also known as exergames, require player movements that would occur in ‘real-life’ exercise participation (Bailey and McInnis 2011) and have been found to be capable of inducing moderate-to-vigorous levels of physical activity (Hwang et al. 2019). Active video games’ potential for exercise promotion is especially relevant during periods of pandemic-related public health restrictions, when access to physical activity facilities is limited (Chtourou et al. 2020).

Active video game platforms, similar to other types of video games, have been evolving along with the development of different types of gaming technologies. For example, although the first virtual reality head-mounted display, Telesphere Mask, came out in 1960 (Heilig 2002), it was not until 2016 that multiple prominent companies started releasing consumer-oriented virtual reality gaming devices that could be used for exercise (Hamilton 2016). We will refer to these virtual reality games that can be used for exercise as *active virtual reality games*, which are considered a subset of active video games.

Both active virtual reality and active video games share a challenge with other video games: difficulty retaining players’ continued interest and engagement (Bailey and Miyata 2019; Chin et al. 2008; Graves et al. 2010; Snow 2011). Most active video games do not have any story plot. Thus, one of the potential solutions to the challenge is to add narratives (Lu et al. 2012; Lu and Kharrazi 2018). Multiple empirical studies have suggested that adding a story to flat screen-based active video games helps to increase players’ physical activity levels (Hwang and Lu 2018; Lu et al. 2016). The story addition may help increase players’ engagement level in those active video games, especially if the narrative portion could be seamlessly transitioned to the active video game play on the same screen. On the other hand, active virtual reality games offer (in general) greater levels of sensory enclosure. Due to such games’ innate ability to provide users with greater levels of presence, there is a question of whether the narrative element would have an impact in their context. To our knowledge, there has been a lack of empirical explorations of narrative element incorporation in active virtual reality games. As it was difficult to find existing active virtual reality games with fully interactive narrative plots, we conducted a feasibility study. We designed this study to determine whether the narrative element incorporation effect could be carried over from a flat screen active video game to a virtual reality gaming environment. We use the term *feasibility study* to indicate a preliminary exploration intended to enable us to prepare for a full-scale study, possibly an intervention (Bowen et al. 2009).

We therefore added a noninteractive narrative animation at the beginning of an existing active virtual reality game that lacked a background story, thus loosely creating a rudimentary narrative version versus the original non-narrative version. We also included a sedentary virtual reality game as a comparison condition and created a narrative versus non-narrative version of that game as well. We wanted to investigate whether incorporating the narrative element, as in its previous flat screen-based research counterparts, could induce greater moderate-to-vigorous physical activity levels in active virtual reality sessions; whether the addition of narratives can improve players’ experience in both active virtual reality and sedentary virtual reality games compared to their non-narrative counterparts; and whether active virtual reality games offer any unique gaming experience when compared to sedentary virtual reality games.

### Sedentary behavior among adults

According to the *US Physical Activity Guidelines* (US Department of Health and Human Services, 2018), adults should engage in 150–300 min of moderate physical activity or 75–150 min of vigorous physical activity each week or an equivalent combination of both types of activities. However, only 23% of the US adult population meet these criteria (Blackwell and Clarke 2018). Insufficient physical activity levels constitute major public health concerns, increasing the risk of multiple diseases (Owen et al. 2011), such as type II diabetes (Hamilton et al. 2014), hypertension (Dempsey et al. 2018), and obesity (Maher et al. 2013). As of 2018, the adult obesity rate was 42.4%, a 26% jump from 2008 (Trust for America’s Health 2020). A recent study found that nearly one in three college-age people in the USA were obese, and more than half were either overweight or obese (Ellison-Barnes et al. 2021). Another study has projected that about half of the US adult population will be obese and about a quarter of them will have severe obesity by the year 2030 (Ward et al. 2019).

During the COVID-19 pandemic, when people were obligated to practice social distancing, they were unable to exercise in public sports facilities, and had only limited opportunities for outdoor activities, the physical activity levels among people significantly decreased around the world (Caputo & Reichert 2020; Dunton et al. 2020). It is noteworthy that, compared to previous generations, the current generation has access to a wide variety of game-based exercise devices at home that they can use during isolation. Preliminary studies have indicated that active virtual reality can help promote the practice of physical activity during the pandemic (Campo-Prieto et al. 2021; Peng et al. 2022). Thus, efforts to encapsulate the power of active screen media such as active virtual reality to increase and motivate physical activity levels are warranted.

### The promise of active video games and virtual reality

Finding engaging methods to increase physical activity is a high-priority task for public health researchers. In 2021, around 80% of those aged 18–35 years in the US spent at least three hours per week playing video games (Entertainment Software Association 2020). Replacing those sedentary gaming hours with physical activity-inducing games would be a potentially viable option for reducing sedentary behaviors. Previous research has shown that active virtual reality games can elicit moderate-to-vigorous physical activity in young adults. For example, Gomez et al. (2018) measured the physiological outcomes of 41 young adults playing three active virtual reality games. They found that active virtual reality games could significantly increase oxygen intake and induce moderate-to-vigorous physical activity, creating an alternative option for physical activity among individuals who are otherwise unmotivated to adhere to traditional physical activity programs. Similarly, Sousa et al. (2022) asked 29 sedentary college students to play a non-narrative active virtual reality game and a sedentary virtual reality game, respectively, and found that the active virtual reality game elicited significantly higher moderate-to-vigorous physical activity levels without an increase in motion sickness (MS) and induced a better game experience when compared to the sedentary virtual reality game.

With the propagation of virtual reality technologies in recent years (e.g., Oculus Quest Series, HTC Vive Pro Series, Valve Index Series), the virtual reality market is enjoying exponential growth, with a projected market value of over $53 billion in 2028 with more ubiquitous individual ownership of the devices (Fortune Business Insights 2021). Virtual reality games may have an immersive edge over other screen-based videogames, given their 360° field of view and vivid audiovisual stimuli, thus making the virtual reality a more likely environment in which players may become lost in their game world (Christensen et al. 2018). Compared to standard screen-based games, a virtual reality game can provide its players with more natural spatial intelligence training by letting them interact with objects situated in a virtual space, thus offers a smoother integration of the player’s movement and exercise (Freina and Canessa 2015). With the development of virtual reality locomotion and the increasingly lighter and ergonomic virtual reality helmets, virtual reality devices will allow more user movement as part of the gameplay, thus making it possible for the gamers to exercise with ease during the play process. Active virtual reality games therefore would become a feasible possibility and differentiate themselves from their mostly sedentary counterparts, sedentary virtual reality games.

As a home-based alternative for physical activity behaviors, active virtual reality offers a potential form of exercise during the COVID-19 pandemic. Similar to traditional active video games, active virtual reality allows players to physically move in a limited living space. Compared to their sedentary counterparts, the “active” component in active virtual reality games can improve the players’ experience by not only providing a virtual task to overcome but also physically challenging the players to achieve their goals. Such achievement can potentially motivate them to increase their physical activity levels to become more active in the game and improve multiple game experience components such as “challenge,” “flow,” and “positive affect,” when compared to sedentary virtual reality games (Sousa et al. 2022).

Although both flat screen-based active video games and active virtual reality can motivate players to exercise through different modes of immersion, the more immersive nature of the virtual reality technologies remains a crucial factor in user experience. In terms of flat screen displays, multiple studies have demonstrated that a larger screen size could enhance presence and participation (Finke et al. 2008; Kim and Sundar 2013; Lin et al. 2006; Lombard et al. 2000). With regard to virtual reality displays, a meta-analysis demonstrated that the display systems employing stereoscopic visuals and wider fields of view are significantly more impactful on user presence (Cummings and Bailenson 2016).

Interestingly, when the different types of display systems are investigated in an active video gaming context, the results seem inconclusive with respect to physical activity behaviors. One study compared the active video gaming experience of a head mounted virtual reality display, a large screen display, and a laptop sized display among 18 participants (Yoo and Kay 2016). Results indicated that, although the virtual reality headset afforded an immersive view field, players perceived the large screen display to be immersive enough and played for longer durations. Another study compared a cycling game shown either via a virtual reality headset or on a laptop display among 37 participants and found that the virtual reality version resulted in a more positive response as well as more caloric expenditure though no statistical test was performed (Amresh and Salla 2017). A recent study compared different active gaming experiences through a head mounted virtual reality display versus a standard flat screen display among 20 participants and found that, although the virtual reality condition resulted in higher moving distance, there was no significant difference between energy expenditure according to the heart rate data (Cao et al. 2021).

Taken as a group, these research projects’ results suggest that, although active video gaming can be carried out in different media platforms with the virtual reality headset potentially providing greater immersion and more engagement, the actual physical activity behavior might not be more pronounced. Thus, these previous findings regarding the addition of a narrative to the flat screen-based games may not implicitly transfer to a virtual reality environment.

### Narrative’s potential

Narratives are one of the most fundamental human characteristics (Barthes 1982) and constitute a fundamental component of game players’ experience and motivation (Yee 2016). Self-determination theory (SDT) scholars have similar theoretical propositions regarding the narrative’s role in enhancing gaming experience by potentially satisfying each of the SDT’s basic needs (Ryan et al. 2006). But, to our knowledge, these propositions have not been theoretically developed or systematically tested. Narratives are pervasive in media and can be defined simply as the occurrence of two or more events in a chronological or causal sequence (Rimmon-Kenan 2002). A more comprehensive definition refers to a narrative as “any cohesive and coherent story with an identifiable beginning, middle, and end that provides information about the scene, characters, and conflict, raises unanswered questions or unresolved conflict; and provides resolution” (Hinyard and Kreuter 2007, p. 778). Characters and plot are the primary narrative components and are important determinants of a narrative’s immersive quality. The characters are a major structural property (Jacobs 2002) and driving force (Surmelian 1969) for a narrative, serving as an internal source of information or beliefs (Green and Brock 2000). The plot, or the “narrative discourse,” is how the story is conveyed. The plot also plays a pivotal role by organizing events into a logically unfolding (often temporal) development of events (Brown 1987; Labov 1972).

Narratives can be powerful in inducing attitude and behavior changes. In recent years, multiple research projects have been published, with increasing exploration of actual behavior change as a result of narrative exposure (Hwang and Lu 2018; Lu et al. 2016; Murphy et al. 2015; Sousa et al. 2020). The most widely demonstrated influence mechanism (Green and Brock 2000), “narrative transportation,” refers to a unique mental process of narrative processing whereby people are absorbed into a story world and become changed by the journey. Transportation is a highly involving and integrative process whereby an individual’s cognitive and affective resources are concentrated. Transportation was originally investigated in textual media with adult participants (Green and Brock 2000), but is now also studied across digital media with participants of all ages. Given the interactive and immersive qualities of games, together with their appeal to children, the term “narrative immersion” was coined from transportation to describe the involving nature of videogames (Sousa et al. 2020).

Suspension of disbelief, reduction of counterarguing, and resistance to persuasion have been explored as additional mechanisms of narrative influence among adults (Moyer‐Gusé and Nabi 2010; Slater and Rouner 2002). Further factors enhancing the narrative impact include making the story experience more personal and creating players’ positive affect to characters through processes such as interpersonal attraction, likeability, identification, and parasocial interaction.

Advances in media technology have enabled narratives to be better woven into games; such advances also could amplify the foundational mechanisms of narrative influence. Video games with effective narrative design could provide an innovative platform that is easy to process, engaging to follow, and fun to experience. Frequent and timely player–character interaction would enhance player engagement with game characters. Well-constructed plots help players move beyond being spectators to become active participants with more agency in the decision-making of the narrative development. As a result, they have extensive control over character and plot development (e.g., the *Walking Dead* series, 2012–2019). Rich scripting using branching logic or Artificial Intelligence can deepen player–character interaction even further. The game characters can be relatable to players and potentially serve as role models (i.e., observational learning). Games with appealing characters and plots can help induce a strong intrinsic motivation for desired behavioral changes. Gameplay experience, integrated with narratives, can facilitate internalization of motivation for behavior changes (e.g., diet and exercise) within and exterior to the game.

Studies have found that adding a narrative to existing flat screen-based active video games can help increase players’ motivation as well as increase physical activity behavior. More specifically, one study added a 3-min animated narrative to an existing Wii active video game and found that children participants in the narrative condition had 40% more objectively-measured steps than their counterparts who played the original game without narratives (Lu et al. 2016). Another study replicated the effect among college students using an active video game on Xbox Kinect (Hwang and Lu 2018). The active video game had the option of switching the narrative mode on and off, with the on setting providing animated comics involving screenshots of themselves to the players. The results indicated that the narrative mode increased moderate-to-vigorous physical activity by 58% compared to the original non-narrative mode. Although promising results suggested positive effects for narrative element incorporation in active video games, whether the effects were due to merely adding a video component or including a narrative was unclear. To rule out the alternative explanation, in another Xbox Kinect console study, two animated videos, one narrative and the other non-narrative, were added to an existing active video game. Results replicated the previous findings: The narrative video resulted in significantly higher immersion than the nonnarrative video. Narrative immersion was positively correlated with moderate-to-vigorous physical activity and average heart rate during gameplay and mediated the effect of the narrative element incorporation on physical activity behaviors (Sousa et al. 2020).

Although narratives have been found to be effective and thus have become a pervasive element for video games on many non-virtual reality platforms, narrative-driven virtual reality games are still relatively rare due to the potential technological difficulty and resources involved in creating effective narratives. For example, a fully immersive virtual reality narrative experience would presume affording the players complete agency of interacting with every element inside the storytelling environment and such type of freedom can be exponentially more expensive to produce. More importantly, such type of freedom is also at odds with the traditional narrative experience, which relies on the author’s didactic control of information delivery. As a result, many industry leaders have acknowledged the challenges brought about by virtual reality technology and argue that alternative and innovative narrative creative strategies must be developed to truly merge virtual reality and narratives (Dredge 2015a, b; Evans 2016; Suduiko 2015).

As a result, it is not surprising that a search of the Steam store, one of the largest digital game distribution platforms in the world (Presser 2022), in May 2022 resulted in 6,351 games tagged with “VR (Virtual Reality)” but the number drastically dwindled to 388, or 6%, when the tag “Story Rich” was also checked (Valve Corporation 2022). While the Steam’s Game Tags are a user generated category, or folksonomy, which is not likely to identify all games with narrative elements, the sharp reduction in the percentage due to the “Story Rich” tag indicates a clear contrast between virtual reality games with a strong narrative presence versus those without.

Moreover, few active virtual reality games, let alone those capable of achieving moderate-to-vigorous physical activity, had narrative elements. Instead, many active virtual reality games simply offer the players instructions on playing the game before leaving them to explore the gaming environment. The key motivational elements in these games include additional levels with elevated points and badges according to the player’s progression, thus likely will be deemed as repetitive by the players over time as such motivational elements do not offer qualitatively new types of information or feedback, and player interest may have plateaued after an extended period. Thus, despite active virtual reality’s potential for physical activity promotion, most active virtual reality games are not designed with sufficiently sustainable motivational elements for the players to continue playing them.

The narrative could be a potential solution to increasing player engagement in virtual reality games. While narratives may have an ending, thus making them not forever lasting, the plot development, when well designed, may offer a more engaging and intriguing grasp on the player’s interests and sustain their play behavior throughout the game. The ideal scenario for narrative element incorporation would be to organically integrate the narrative into the game development and allow players to interact with playable and non-playable characters inside the story throughout the gameplay and alter the narrative development over their interactions. Such type of fully immersive, story-rich virtual reality products may still be relatively difficult to create due to various technological and medium constraints (Bailenson 2018). However, preliminary studies showed that even barebone narratives, introduced merely as sound clips, may foster players’ engagement when added to a fully immersive virtual reality environment. For example, Weech et al. (2020) found that playing the players’ enriched audio narratives helped increase presence and reduced cybersickness, especially among non-gamers. Aside from sound clips, film clips offer additional visual elements on top of the auditory medium and can serve as a feasible modal for narrative incorporation in virtual reality environments. As with flat screen-based active video games, the incorporation of narratives into virtual reality games could help make the players’ experience more engaging and enjoyable, and potentially elevate the long-term adherence to it (Lu 2015).

The effect of the narrative element incorporation in active virtual reality games on moderate-to-vigorous physical activity has not yet been empirically tested. This could be a key element to increasing players’ interest in active virtual reality and hallmarking active virtual reality as a feasible and interesting option to increase physical activity levels and game engagement in a home environment. More importantly, the test of a narrative within a virtual reality environment will extend the narrative’s motivational effect beyond the previous non-virtual reality context.

We investigated the effect of narrative element incorporation on gameplay and physical activity behavior (mainly for active virtual reality) as a feasibility study for a possible future intervention. We also studied the differential effect of the type of virtual reality game (active virtual reality vs. sedentary virtual reality) on game experience and physical activity enjoyment (PAE). We employed an existing active virtual reality game versus a sedentary virtual reality game and created non-interactive narrative animations for both games. As both games were commercial off-the-shelf versions, thus they did not allow us to modify the opening scenes to include the narrative animations, we decided to play the animations on a flat screen display before asking the players to switch to the virtual reality headset display.

Our main hypothesis is that adding narratives will increase players’ gameplay duration and improve their game experience for both active virtual reality and sedentary virtual reality. Active virtual reality with narratives would also induce higher moderate-to-vigorous physical activity levels than non-narrative active virtual reality. In addition, active virtual reality would elicit a better game experience and higher players’ physical activity engagement than sedentary virtual reality without a significant increase in motion sickness (MS).

## Materials and methods

### Ethical concerns

The institutional review board at the first author’s institution approved this study. All participants signed a written informed consent and were provided with a copy.

### Recruitment

We invited participants via web advertisements and posters displayed on campus in the first author’s institution. Since less than a quarter of the US adult population participate in sufficient physical activities (Blackwell and Clarke 2018) and those with low levels of physical activity are more likely to continue to be sedentary in later life (Varma et al. 2017), we believe that people who are not active could benefit the most from the effect of an alternative option to increase physical activity levels with some potential for long-term engagement through active virtual reality and narratives, especially during the pandemic. Therefore, given the university setting, our inclusion criteria were that participants should be between 18 and 29 years of age; have low levels of physical activity (defined as a weekly reported total physical activity Metabolic Equivalent of Task (MET) minutes ≤ 1500 min as calculated using the International Physical Activity Questionnaire–Short Version) (IPAQ-SV) (Booth 2000); have no cardiovascular diseases, cerebrovascular diseases, neurological diseases, attention disorders, or physical disabilities; and speak English. We believe that this population is more likely to capture evidence of the effect induced by narratives. To ensure that the prior knowledge about the current games did not confound the results, we decided only to invite those who have never played the virtual reality games used in this study.

A total of 252 students indicated their interest through an online pre-screening process by completing questionnaires, including the IPAQ-SV. Most of the respondents (184, 73%) were excluded due to high levels of self-reported physical activity (161, 64%) or prior experience (23, 9%) with the virtual reality games selected for this study, leaving 68 who met our inclusion criteria. All ineligible students received a “Thank you” e-mail for their interest in our project. The remaining 68 individuals were invited to the initial screening at the beginning of the first visit. Of the 68, 45 (66%) completed both visits in an on-campus research laboratory from October 2020 to March 2021. The rest of the 23 students did not show up for at least one of their visits due to contracting COVID-19 or scheduling conflicts. Of the 45 students who completed the data collection, eight were excluded due to technical difficulties resulting in nonvalid data (e.g., the virtual reality game crashed multiple times during one play session and took a long time to fix before it restarted normally). Only one research assistant (RA) was allowed in the space during data collection due to social distancing requirements, which prevented simultaneously monitoring whether all devices were working properly. One participant was excluded due to falling asleep during the second visit’s questionnaire session. Thus, our final sample size consisted of 36 participants.

### General procedures

After signing the informed consent, participants entered their demographic information in an online questionnaire during their first visit. We then randomized the participants into either the narrative or non-narrative conditions (this constituted our between-subject factor). Our RAs measured the participants’ height using a stadiometer (ShorrBoard, Weight and Measure, LLC, Olney, MD, USA) and measured their weight using a calibrated scale (SECA 813, SECA Inc., Chino, CA, USA). Each participant visited the lab twice and was asked randomly to play either an active virtual reality or a sedentary virtual reality game during each visit (as stated previously, this was our within-subject factor). The visits were scheduled so that there were 48–120 h between them to permit participants to rest between sessions.

An RA first provided participants with instructions for playing the active virtual reality game or the sedentary virtual reality game during each visit. We collected participants’ mobile devices to ensure their attention would be entirely focused on the study sessions. The RA then attached an accelerometer (GT9X Link; ActiGraph, Pensacola, FL, USA) to the participant’s non-dominant wrist and informed the participant that next they would play a virtual reality game and answer some questions afterwards.

More specifically, the participants in the non-narrative condition went directly to playing the virtual reality games. If the participant was in the narrative condition, the RA asked them to sit in front of a high-definition television connected to a desktop computer (PC), which had a 5-min narrative video preloaded using a multimedia player program. The RA then pressed “Play” on the PC to launch the video. Immediately after the narrative video ended, the RA guided the participant to a nearby space in the lab to put on a virtual reality headset (usually an optimal adjustment took less than 15 s to achieve) before launching the game. Two additional spaces were set aside in the lab for use by study participants: (1) a space where participants could move around as they needed to do without any obstacles to play the active virtual reality game; (2) a space with a chair for participants to sit in to play the sedentary virtual reality game. The two gameplay spaces and the narrative viewing area were adjacent to each other thus walking from one space to another required fewer than 3 s. To ensure a seamless transition for the participants in the narrative condition, no additional instructions were given between the end of the narrative video and the start of the virtual reality gameplay sessions (a gap of approximately 10–18 s). In addition, during the virtual reality headset adjustment phase, the RA only interacted minimally with the participants in both non-narrative and narrative conditions, generally to ensure that the virtual reality games loaded correctly, and that the headset as well as the controllers worked properly.

We instructed them that they could play as much as they wanted, though the RA would stop them if they played for more than 60 min to avoid exhaustion, especially for the active virtual reality condition (Crewe et al. 2008). At the end of each session, participants completed the Game Experience Questionnaire (GEQ) (IJsselsteijn et al. 2013) along with several other questionnaires: PAE (Kendzierski and DeCarlo 1991), Motion Sickness (MS) (Liang and Lin 2018), and Social Desirability (SD) (Reynolds and Paget 1983). Self-report instruments were randomized at the item level and blocked at the instrumental level. Participants who completed both visits received a $30 gift card as our team’s appreciation for their time and effort. They were then thanked and left with a copy of their consent form.

### VR: active virtual reality versus sedentary virtual reality games

We used the HTC Vive Pro system (HTC, New Taipei, Taiwan) for both virtual reality sessions. The Vive Pro headset is fairly lightweight (803 g). It features high-resolution (1440 $\times$ 1600 pixels per eye) displays with a second outward-facing camera, dual active noise-canceling microphones, and a wireless adapter for untethered play.

**Fig. 1:**
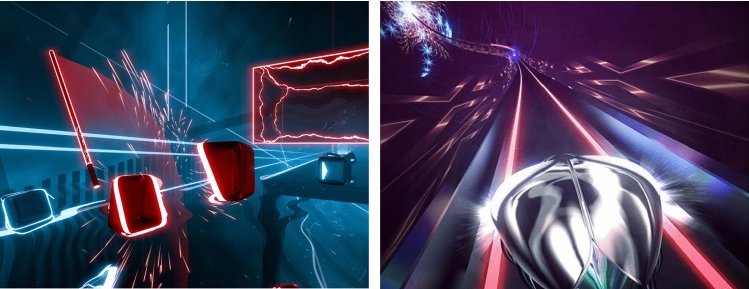
Screenshots of the virtual reality games (left, the active game, *Beat Saber*; right, the sedentary game, *Thumper*). Game notes: *Beat Saber*, developed by Beat Games; © Beat Games 2019. *Thumper*, developed by Drool; © Drool 2016

The active virtual reality and sedentary virtual reality games selected for this study are *Beat Saber* (Beat Games 2019) and *Thumper* (Drool 2016), respectively. Both of them are rhythm games with similar visual and auditory characteristics and have received generally positive reviews years after their release (Mitchell 2022), though they differ significantly in the amount of physical activity required to play (Sousa et al. 2022). *Beat Saber*, the active virtual reality game, is a rhythm-based game in which the participant uses virtual lightsabers in the form of laser swords to cut through small blocks floating in different directions (incurring primarily upper-body movements) and evades large blocks by moving to either side of them or by crouching under them (incurring primarily whole-body movements). All of the blocks would constantly move swiftly in the player’s direction in different patterns according to the beats of the background music. *Thumper*, the sedentary virtual reality game, is also a rhythm-based game in which the participant uses an Xbox One controller to direct a beetle-like character to travel through different worlds consisting of different shapes of colorful tracks across the galaxy by moving only their fingers. By hitting on increasingly fast incoming “notes” on the track in synchrony with background music, the participant can guide the beetle to avoid obstacles, turn against curved walls, and ultimately move on to the next levels. See Fig. 1 for their respective screenshots.

We recruited all participants as first-time players of *Beat Saber* and *Thumper* to reduce the familiarization effect. To avoid the games’ difficulty curve potentially thwarting their continued attempt for gameplay, for *Beat Saber*, we created a song list that began with an easily paced song to ensure the participant could fully grasp the game control and the game instructions. The difficulty level would then gradually increase with each subsequent song. The playlist was as follows: *USD100*; *Country Roads*; *Escape*; *Beat Saber*; *Level Insane*; *Turn Me On*; *Elixia*; *Commercial Pumping*; *Unlimited Power*; and *Balearic Pumping*. For *Thumper*, the levels sequentially increase in difficulty and thus no further modification was required.

### Narrative videos

As neither *Beat Saber* nor *Thumper* has any story elements in their original forms, we created narratives for both games after consulting a group of expert virtual reality game players and Sci-Fi fans who are familiar with both games and popular Sci-Fi game and movie narratives. Given the cost associated with creating 3D animation narratives, we decided to use clips from existing video productions and edit them into trailer-like movies broadcast before the gameplay. To ensure visual and thematic consistency between the animation and the games, multiple hours of narrative 3D animated videos containing similar characters, scenarios, location settings, and atmospheres that resembled each virtual reality game were first downloaded as high-definition clips from online video sharing sites. They include a range of different Sci-Fi movies and game clips, from esoteric to popular titles. Then, the same group of students and experts went through each of the videos and worked out multiple potential editing sequences that would result in new stories that serve as opening animations for either of the games. The team then worked with a professionally trained student video editor to help create different narrative montages of new stories. The group worked closely with the editor over multiple iterations of refining the editing and transitioning of scenes to ensure that the videos were engaging and would integrate well with both virtual reality games.

Given the visual similarity of the saber in the *Beat Saber* and the lightsabers in the *Star Wars* universe, the narrative is set in a *Star Wars* universe where the player is among a new generation of apprentices training to use a lightsaber to disperse the dark force. The *Thumper* narrative is based on a similar futuristic premise involving space travel where the player is recruited as part of a special force for a pioneering space expedition for the survival of the entire human race. Each video was edited to be around five minutes in length, ensuring sufficient time for story engagement. Each video ends with a cliffhanger-like scene before encouraging the players to play the active virtual reality or sedentary virtual reality game afterward. At the end of both videos, the screen went dark, then a call-to-action type of script was displayed (*Beat Saber*: “Your training begins now”; *Thumper*: “Embark on your adventure.”) along with the opening scene in each game title.

### PA assessment

ActiGraph accelerometer devices are widely used to assess young adults’ levels of physical activity in various gaming environments (Hwang et al. 2018; McDonough et al. 2018). In this study, we attached an ActiGraph GT9X Link to each participant’s non-dominant wrist and initialized the device at an 80-Hz sampling rate. The ActiGraph triaxial accelerometers assessed physical activity by providing acceleration measures from the participant’s movement along three axes (anterior–posterior, vertical, and medial–lateral). Thus, we obtained the frequency, duration, and intensity of the participants’ physical movements (Strath et al. 2013). Additionally, when we processed the physical activity data, we excluded the narrative group’s movement from the 5-min video watching process to ensure that both groups were compared only for their active virtual reality gameplay behaviors.

Due to the COVID-19 pandemic physical distancing requirement, given the laboratory’s dimension, only one RA could be in the space with the participant, which significantly limited our ability to detect equipment malfunctions promptly. The wrist-worn ActiGraph GT9X Link data were downloaded and converted via ActiLife (Version 6.13.2; ActiGraph, Pensacola, FL, USA) to a triaxial form to obtain activity counts, which we used to quantify the amplitude and frequency of detected accelerations at a one-second epoch. We chose this epoch level because the one-second epoch is the appropriate epoch length to detect and examine short bursts and intermittent physical activity behaviors (Hwang et al. 2018). We adapted the established adult cut-off points to estimate the time spent in light, moderate, and vigorous physical activity: light < 760 counts/min; moderate = 760–5724 counts/min; vigorous > 5724 counts/min (Freedson et al. 1998; Matthew 2005). We also originally used a hip-worn accelerometer on all participants (wGT3x-BT, ActiGraph, Pensacola, FL, USA), but discarded that data due to malfunctioning equipment that did not accurately register participants’ movements. For example, we found significantly lower correlations between hip and wrist accelerometer measures (*r* = 0.08, n.s.) while measures from previous similar projects tend to have significantly higher correlations (*r* = 0.80–0.95, *p*s < 0.05) (Hwang et al. 2018).

### Questionnaires: GEQ, PAE, MS, and SD

After each active virtual reality or sedentary virtual reality session, participants completed the GEQ (IJsselsteijn et al. 2013), PAE (Kendzierski and DeCarlo 1991), MS (Liang and Lin 2018), and SD (Reynolds and Paget 1983) questionnaires. We used the original GEQ (IJsselsteijn et al. 2013) scale with 33 items, measuring seven components of the game experience, including competence (e.g., “I felt skillful.”), sensory and imaginative immersion (e.g., “It felt like a rich experience.”), flow (e.g., “I lost track of time.”), tension (e.g., “I felt frustrated.”), challenge (e.g., “I thought it was hard.”), and positive (e.g., “I felt content.”) and negative affect (e.g., “I felt bored.”). Each item had a 5-point Likert-style scale response (0 = *not at all*; 4 = *extremely*). The PAE (Kendzierski and DeCarlo 1991) questionnaire was a 16-item scale assessing the enjoyment of physical activities, which is a crucial construct of adherence to physical activity behaviors (Wankel 1993). A sample question is “(1 =) (Physical activity) is no fun at all” versus (5 =) (Physical activity) is a lot of fun.” The MS (Liang and Lin 2018) questionnaire was a 15-item, 10-point Likert-style scale (0 = *not at all*; 9 = *severely*) assessing the level of feeling discomfort brought on by moving in a virtual reality environment (sample question: “I felt queasy.”). The 9-item SD (Reynolds and Paget 1983) questionnaire was included to control for demand characteristics (1 = *disagree*; 5 = *agree*) to ensure that participants were not answering questions to please the research team; sample questions included, “I tell the truth every single time,” “I never say things I shouldn’t,” and “I never lie.”

### Statistical analysis

We scored and analyzed all the GEQ-derived variables following a recommended approach (IJsselsteijn et al. 2013). The GEQ indicated good reliability after both game sessions (Active virtual reality: overall Cronbach’s *α* = 0.87; Sedentary virtual reality: overall Cronbach’s *α* = 0.93), as did the PAE (Active virtual reality: Cronbach’s *α* = 0.93; Sedentary virtual reality: Cronbach’s *α* = 0.96); MS (Active virtual reality: Cronbach’s *α* = 0.95; Sedentary virtual reality: Cronbach’s *α* = 0.93) and the SD (Cronbach’s *α* = 0.84 for all).

We ran general linear models with two factors (two-way ANOVA) with “game” (Within subject: Active virtual reality vs. Sedentary virtual reality) and “condition” (Between subject: narrative vs. non-narrative) as random factors (Gelman 2005). We also applied a multivariate model adjusted for SD with “game” as the independent factor (Active virtual reality vs. Sedentary virtual reality) to test its effect on PAE, GEQ, and MS. We used the partial eta square (*η*^2^_p_) to estimate the effect size. Bonferroni’s post hoc was used to identify specific differences among the sessions.

## Results

Power calculations were conducted using G*Power (Version 3.1) (Faul et al. 2009). We have decided to refer to prior research that reported an examination of the narrative impact on physical activities as the foundation for the effect size estimation (Hwang and Lu 2018; Lu et al. 2016; Sousa et al. 2020). Although the range for the f statistics was between 0.3 and 0.57 with an average of 0.425, we used a slightly more conservative estimate of 0.333. The post hoc power calculation (Onwuegbuzie and Leech 2004) using our actual sample size (*n* = 36) for our primary analysis (the within-between analysis with two random independent factors) indicated we had sufficient power to detect an effect size of *η*^2^_p_ = 0.1 (critical *F* = 4.13), and our analysis was set at a power of 97%. The significance level was set at *p* = 0.05. All other statistical procedures were performed using SPSS (Version 26.0; IBM Corp., Armonk, NY, USA).

Of the 36 participants, 25% were women (*n* = 9) and 75% were men (*n* = 27). The randomized groups did not have statistically significant differences regarding their sex (proportion), age, height, weight, BMI, or SD (Table 1).

**Table 1:** Participant demographic and anthropometric characteristics (*N* = 36)

Characteristics	Narrative condition (*n* = 18)	Non-narrative condition (*n* = 18)	*p* value
*Sex, n (%)*			
Female	3 (16.7)	6 (33.3)	0.26
Male	15 (83.3)	12 (66.7)	
Age (years)	23.0 ± 2.9	23.3 ± 2.7	0.74
Height (cm)	174.8 ± 9.0	175.0 ± 8.4	0.96
Weight (kg)	73.6 ± 15.0	71.0 ± 17.6	0.63
BMI (kg/m^2^)	24.0 ± 3.9	23.1 ± 5.1	0.57
SD (score)	2.98 ± 0.81	3.20 ± 0.75	0.40

*BMI* body mass index, *SD* social desirability

Overall gameplay (minutes) did not show significant effects for “Active virtual reality versus Sedentary virtual reality” game condition (Active virtual reality: 26.3 ± 11.6/ Sedentary virtual reality: 27.1 ± 16.9; F = 0.09, *p* = 0.77, η_p_^2^ < 0.01) or for the “narrative versus non-narrative condition” (Active virtual reality: 24.9 ± 11.2 vs. 27.8 ± 12.2 / Sedentary virtual reality: 26.3 ± 17.1 vs. 27.8 ± 17.1; F = 0.26, *p* = 0.61, η_p_^2^ = 0.01) (Table 2). Despite a trend of difference, the “narrative versus non-narrative” condition did not show statistically significant effects on minutes of non-movement (F = 1.09, *p* = 0.30, η_p_^2^ = 0.03), minutes of light physical activity, (F = 0.10, *p* = 0.76, η_p_^2^ < 0.01), or minutes in moderate-to-vigorous physical activity (F = 0.89, *p* = 0.35, η_p_^2^ = 0.03). Participants in the narrative condition spent an average of 14.2 ± 5.5 min in absolute moderate-to-vigorous physical activity, whereas those in the non-narrative condition spent 12.2 ± 7.3 in moderate-to-vigorous physical activity minutes when playing the active virtual reality.

**Table 2:** General linear model results (expressed as mean and standard deviation) for gameplay duration, non-movement, light PA, and MVPA for active virtual reality (AVR) versus sedentary virtual reality (SVR) games, with and without narrative

	Narrative condition	Non-narrative condition	Game effect (AVR vs. SVR; within)	Condition effect (narrative vs. non-narrative; between)	Interaction (game x condition)
*Gameplay duration (min)*			F = 0.09	F = 0.26	F = 0.09
AVR game 26.3 ± 11.6	24.9 ± 11.2	27.8 ± 12.2	*p* = 0.77	*p* = 0.61	*p* = 0.77
SVR game 27.1 ± 16.9	26.3 ± 17.1	27.8 ± 17.1	η_p_^2^ < 0.01	η_p_^2^ = 0.01	η_p_^2^ < 0.01
*Non-movement (min)*			**F = 57.03**	F = 1.09	F = 0.17
AVR game 6.3 ± 7.4	4.1 ± 2.5	8.5 ± 9.8	***p* < 0.01**	*p* = 0.30	*p* = 0.68
SVR game 25.7 ± 15.7	24.5 ± 15.0	26.9 ± 16.8	**η**_**p**_^**2**^** = 0.63**	η_p_^2^ = 0.03	η_p_^2^ = 0.01
*Light PA (min)*			**F = 60.34**	F = 0.10	F = 0.14
AVR game 6.8 ± 4.7	6.5 ± 4.4	7.1 ± 5.0	***p* < 0.01**	*p* = 0.76	*p* = 0.71
SVR game 0.6 ± 0.7	0.6 ± 0.9	0.5 ± 0.5	**η**_**p**_^**2**^** = 0.64**	η_p_^2^ < 0.01	η_p_^2^ < 0.01
*MVPA (min)*			**F = 144.48**	F = 0.89	F = 0.93
AVR game 13.2 ± 6.5	14.2 ± 5.5	12.2 ± 7.3	***p* < 0.01**	*p* = 0.35	*p* = 0.34
SVR game 0.4 ± 0.6	0.4 ± 0.8	0.4 ± 0.4	**η**_**p**_^**2**^** = 0.81**	η_p_^2^ = 0.03	η_p_^2^ = 0.03
*Non-movement (%)*			**F = 889.08**	**F = 4.72**	**F = 5.32**
AVR game 22.0 ± 16.1	16.2 ± 6.8	27.8 ± 20.4	***p* < 0.01**	***p* = 0.04**	***p* = 0.03**
SVR game 96.3 ± 3.3	96.3 ± 3.9	96.4 ± 2.7	**η**_**p**_^**2**^** = 0.96**	**η**_**p**_^**2**^** = 0.12**	**η**_**p**_^**2**^** = 0.14**
*Light PA (%)*			**F = 204.73**	F < 0.01	F = 0.03
AVR game 25.4 ± 9.7	25.2 ± 6.9	25.6 ± 12.1	***p* < 0.01**	*p* = 0.97	*p* = 0.87
SVR game 2.1 ± 1.9	2.2 ± 2.2	2.0 ± 1.5	**η**_**p**_^**2**^** = 0.86**	η_p_^2^ < 0.01	η_p_^2^ < 0.01
*MVPA (%)*			**F = 330.68**	**F = 4.24**	**F = 4.64**
AVR game 52.4 ± 17.8	58.4 ± 12.3	46.4 ± 20.7	***p* < 0.01**	***p* = 0.05**	***p* = 0.04**
SVR game 1.6 ± 1.6	1.5 ± 1.8	1.6 ± 1.3	**η**_**p**_^**2**^** = 0.98**	**η**_**p**_^**2**^** = 0.11**	**η**_**p**_^**2**^** = 0.12**

*PA* physical activity, *MVPA* moderate-to-vigorous physical activity

Significant effects are bolded

**Fig. 2:**
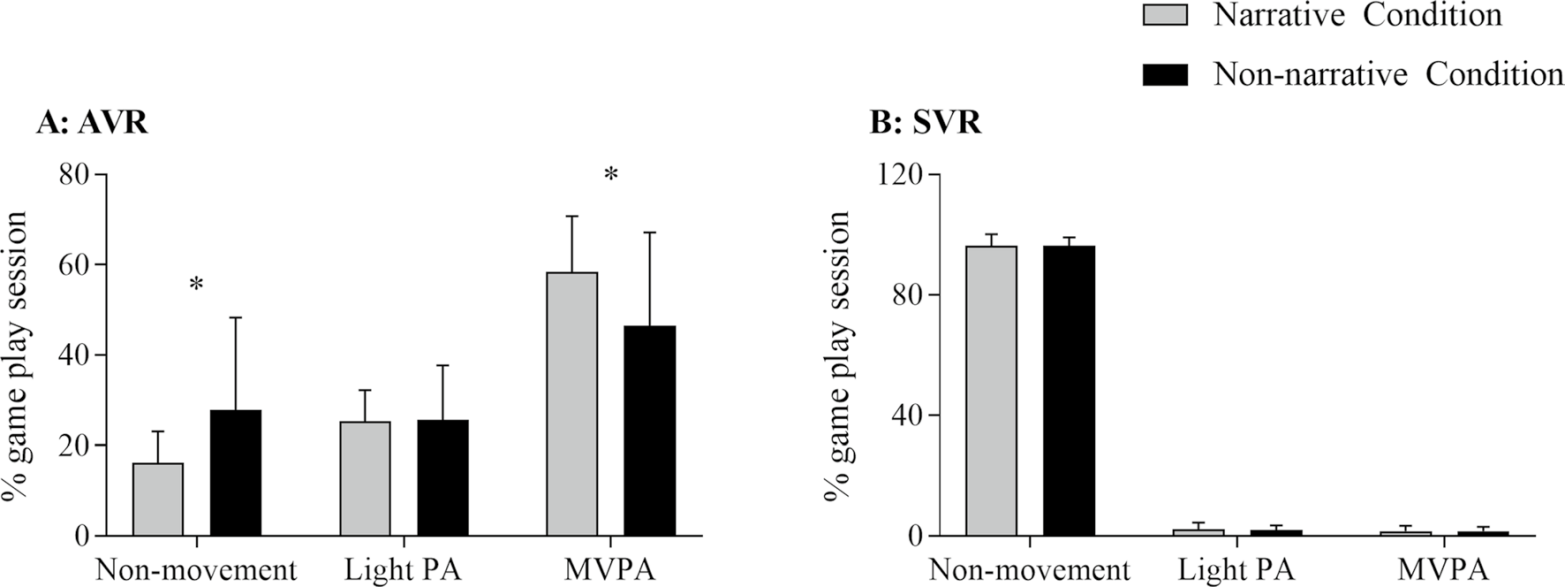
Percent (%) of time spent in non-movement, light PA, and MVPA during the active virtual reality (AVR; **a**) and the sedentary virtual reality (SVR; **b**) game sessions, with and without narrative. * = Statistically significant difference between narrative versus non-narrative. Abbreviations: *PA* physical activity, *MVPA* moderate-to-vigorous physical activity, *VR* virtual reality

However, when considering the relative values of different types of physical activity (calculated as the percentage of a specific activity duration divided by the overall gameplay duration) within each session, we have found that the participants under the narrative condition in the active virtual reality sessions spent relatively less time (expressed in %) in non-movement (Narrative: 16.2 ± 6.8 vs. Non-narrative: 27.8 ± 20.4; F = 5.32, *p* = 0.03, η_p_^2^ = 0.14) and more time (%) in moderate-to-vigorous physical activity (Narrative: 58.4 ± 12.3 vs. Non-narrative: 46.4 ± 20.7; F = 4.64, *p* = 0.04, η_p_^2^ = 0.12) than the non-narrative condition. No differences were identified for relative value of light physical activity (%) (Narrative: 25.2 ± 6.9 vs. Non-narrative: 25.6 ± 12.1; F = 0.03, *p* = 0.87, η_p_^2^ < 0.01) (See Table 2, Fig. 2 for details). Please note that the overall gameplay duration in the narrative condition had excluded the initial 5-min spent watching narratives.

**Fig. 3:**
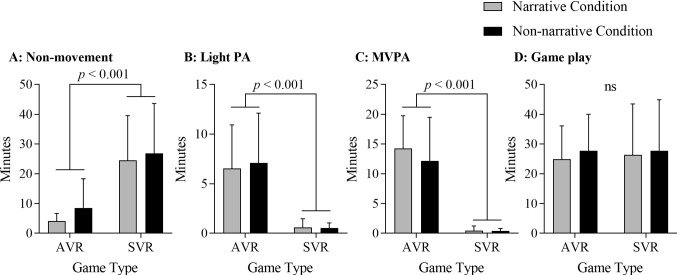
Minutes of non-movement, light PA, MVPA, and gameplay during the active virtual reality (AVR) and sedentary virtual reality (SVR) game sessions, with and without narrative. ns = non-significant. Abbreviations: *PA* physical activity, MVPA moderate-to-vigorous physical activity, *VR* virtual reality

As expected, the results of our GLM in which we tested the “game” and “condition” effect over minutes of non-movement, light physical activity, and moderate-to-vigorous physical activity indicated that the type of “game” had a statistically significant effect on non-movement (Active virtual reality: 6.3 ± 7.4 vs. Sedentary virtual reality: 25.7 ± 15.7; F = 57.03, *p* < 0.01, η_p_^2^ = 0.63), light physical activity (Active virtual reality: 6.8 ± 4.7 vs. Sedentary virtual reality: 0.6 ± 0.7; F = 60.34, *p* < 0.01, η_p_^2^ = 0.64), and moderate-to-vigorous physical activity (Active virtual reality: 13.2 ± 6.5 vs. Sedentary virtual reality: 0.4 ± 0.6; F = 144.48, *p* < 0.01, η_p_^2^ = 0.81). Similarly, in terms of the relative values of different types of physical activity (%), the type of “game” had statistically significant effect accordingly: non-movement (Active virtual reality: 22.0 ± 16.1 vs. Sedentary virtual reality: 96.3 ± 3.3; F = 889.08, *p* < 0.01, η_p_^2^ = 0.96), light physical activity (Active virtual reality: 25.4 ± 9.7 vs. Sedentary virtual reality: 2.1 ± 1.9; F = 204.73, *p* < 0.01, η_p_^2^ = 0.86), and moderate-to-vigorous physical activity (Active virtual reality: 52.4 ± 17.8 vs. Sedentary virtual reality: 1.6 ± 1.6; F = 330.68, *p* < 0.01, η_p_^2^ = 0.98) (See Table 2, Fig. 3 for details).

The multivariate model to test the “game” effect on the GEQ, PAE, and MS components indicated a statistically significant game effect favoring active virtual reality for flow, positive affect, and PAE over sedentary virtual reality. After we corrected the model for SD, the positive affect and PAE effects remained significant, but the flow effect did not (Table 3). We found, for the GEQ, a significant “game” and borderline “game × condition” interaction effect for Competence (Game: Active virtual reality: 3.1 ± 1.0 vs. Sedentary virtual reality: 3.1 ± 0.9; F = 6.01, *p* = 0.02, η_p_^2^ = 0.15; Interaction: F = 2.92, *p* = 0.10, η_p_^2^ = 0.08), and a borderline interaction effect for SII (F = 3.28, *p* = 0.08, η_p_^2^ = 0.09). No significant effects were found for the other GEQ components (See Table S1 for details). MS was low and not significantly different across conditions (Means range: 1.5–2.24, *p*s > 0.2).

**Table 3:** Multivariate model results for the Game Engagement Questionnaire (GEQ) measures, PAE, and MS, expressed as mean and standard deviation, for active virtual reality (AVR) versus sedentary virtual reality (SVR) games, before and after Social Desirability adjustment

	Game session		Game effect (Within) (adjusted by SD)		
	AVR	SVR	F	*p *value	Effect size (η_p_^2^)
GEQ—competence	3.1 ± 1.0	3.1 ± 0.9	0.06 (0.05)	0.81 (0.95)	0.01 (0.02)
GEQ—Sensory and imaginative immersion	3.2 ± 0.8	3.0 ± 1.0	0.72 (0.92)	0.40 (0.40)	0.01 (0.03)
GEQ—flow	3.7 ± 0.8	3.2 ± 1.0	**4.27 (2.26)**	**0.04 (0.11)**	**0.06 (0.06)**
GEQ—tension	1.4 ± 0.6	1.6 ± 0.8	1.15 (0.59)	0.29 (0.56)	0.02 (0.02)
GEQ—challenge	2.7 ± 0.8	2.4 ± 0.8	3.49 (2.21)	0.07 (0.12)	0.05 (0.06)
GEQ—negative affect	1.8 ± 0.8	1.6 ± 0.5	1.07 (1.40)	0.31 (0.25)	0.02 (0.04)
GEQ—positive affect	3.8 ± 1.0	3.2 ± 1.1	**6.74 (3.67)**	**0.01 (0.03)**	**0.09 (0.10)**
PAE	4.2 ± 0.6	3.7 ± 0.8	**9.61 (4.81)**	** < 0.01(0.01)**	**0.12 (0.12)**
MS	1.9 ± 1.4	1.7 ± 1.3	0.26 (0.56)	0.61 (0.57)	< 0.01(0.02)

*PAE* physical activity engagement, *MS* motion sickness

Significant effects are bolded

## Discussion

To investigate the effect of narrative element incorporation on physical activity and game experience, we created two five-minute noninteractive 3D animation clips to be integrated into an active virtual reality and a sedentary virtual reality game. We found that the narrative element incorporation in active virtual reality induced a significantly higher percentage of moderate-to-vigorous physical activity and a significantly less amount of time spent in non-movement when compared to the non-narrative version of the active virtual reality. This result partially supported our hypothesis that the narrative element incorporation could increase moderate-to-vigorous physical activity during active virtual reality, even though the absolute time spent in moderate-to-vigorous physical activity and overall gameplay duration did not increase with narrative element incorporation. We have also found that active virtual reality elicited a better game experience and PAE than sedentary virtual reality without a significant increase in MS, which supported our hypothesis regarding the active virtual reality versus sedentary virtual reality comparison.

A recent examination of the comparison between active virtual reality versus sedentary virtual reality sessions suggested that active virtual reality elicited significantly higher moderate-to-vigorous physical activity and borderline more cognitive enhancement among sedentary college students (Sousa et al. 2022). Our findings replicated the moderate-to-vigorous physical activity findings in that study and further demonstrated that active virtual reality elicited a better game experience with higher positive affect and PAE than sedentary virtual reality. This suggested active virtual reality’s potential for positive player engagement and sedentary behavior reduction. This finding is noteworthy for long-term adherence to active virtual reality interventions and physical activity promotion in general since a positive experience when engaging in physical activity has been found to be key for sedentary behavior reduction and long-term adherence to physical activity intervention (Parschau et al. 2014; Rose and Parfitt 2012).

A narrative can elicit transportation, a phenomenological experience in which people “travel” into the story world and are transformed by the journey (Green 2004). The narrative element incorporation can potentially immerse the players in a story, thereby eliciting more engagement with the game and a higher proportion of time spent in moderate-to-vigorous physical activity behavior without increasing the overall gameplay duration. We demonstrated preliminary evidence for the effect of the narrative element incorporation when the narrative was integrated with active virtual reality games compared to the non-narrative counterpart. The lack of support for absolute moderate-to-vigorous physical activity duration and overall play duration hypotheses could be partially explained by several factors.

First, the sample size adopted in this study might lack the statistical power required to detect small and moderate effect sizes. Thus, the negative results of this study should be interpreted with reasonable caution. Due to the novelty of this research in terms of design and measurement, we could not identify from the active virtual reality literature articles that specifically address the exact outcome presumed effect sizes. However, we were able to use prior work to derive a solid justification for our chosen effect size for power analysis. We hope this study will help pave the path for future studies with more participants and more comparative conditions.

In terms of the narrative clips, unlike those from previous flat screen-based active video game studies that resulted in more significant results in absolute duration (Hwang et al. 2019; Lu et al. 2016; Sousa et al. 2020), the story videos used in this study were not created by professional media and game production companies specifically for the games or for the audiences. Instead, they consist of a compilation of pre-existing videos made by various artists, which may have limited the immersive appeal. Additionally, we chose a *Star Wars*-type of background story for the active virtual reality game mainly due to the visual similarities of the lightsabers. However, the study was completed more than forty years since the series 1977 original release, with the audience less enthusiastic toward the movie series over time (Rottentomatoes.com 2019). The so-called *Star Wars Franchise Fatigue* has been discussed extensively by critics as well as an actor in the series (Burwick 2019; Rubin 2022). Therefore, the overall sentiment toward another *Star Wars* stories among the participants might not be enthusiastic to begin with and could further curb their interest and potential engagement-related behavior. Such adverse sentiments could be avoided in the future if audience studies can be conducted before the study takes place to ensure the narrative would be interesting and appealing to the players.

The duration of the narrative video (~ 5 min) and its screening on a TV before the virtual reality gameplay may also be insufficient for the virtual reality environment due to the modality shift as well as lack of the virtual reality consistency since other story-rich virtual reality games tend to scatter narrative segments throughout the gameplay and allow many interactive segments to retain the players’ interest within the virtual reality environment. Thus, our study design may have suffered from low ecological validity. We suggest that future research should sample or create stimuli in which the narrative and designated gameplay blocks do not vary in their immersive qualities and maintain both the narrative display and game play on the same platform. Besides, due to our feasibility study’s design intended to imitate a truly interactive narrative virtual reality play session, we did not get a chance to measure players’ narrative evaluation immediately after their viewing session to assess their attitude toward the narratives. Future studies might add qualitative data collection after the players have completed the play session to have some in-depth interviews with the players about their opinions of the appeal of the narrative and the virtual reality games.

We need to report one final factor that may have caused us some difficulties. We conducted this study during the COVID-19 pandemic with the aim of exploring exercise options during the pandemic. Despite that the research team tried their best to ensure physical distancing and thorough sanitation of the research space and device after each play session, the participants might also have concerns about exercising vigorously in a closed space for an extended period. Similarly, while the RA wore a mask throughout the data collection, this might also signal to the participants that this was not a typical virtual reality play session at home. As a result, they might have reduced their level of physical exhaustion to avoid excessive air exchange, which led to a lack of significant findings on the physical activity behaviors. Nevertheless, both conditions were affected equally during the pandemic, and we still managed to find some differences. Again, future in-depth interviews at the end of the study will shed light on a more nuanced interpretation of the player's motivation and behavior. Additionally, we would like to expand the current project when the COVID-19 pandemic restrictions are fully lifted and students have returned fully to their pre-pandemic habits to explore potential behavioral differences between then and our current study.

Despite these limitations regarding narrative production, delivery, and research setting, we still observed that the participants played the active virtual reality more intensely (with a higher proportion of moderate-to-vigorous physical activity and lower proportion of non-movement) after watching a five-minute noninteractive narrative before the play sessions. Our results suggested that this simple manipulation of the rudimentary narrative delivery before the play sessions might have slightly increased players’ engagement to elevate the relative moderate-to-vigorous physical activity duration but was insufficient to increase the absolute moderate-to-vigorous physical activity duration and overall gameplay duration.

We are among the first to empirically explore the effect of narrative element incorporation on fully immersive virtual reality games. The results suggested that there might be some boundary conditions to the narrative effect. More specifically, narrative production, duration, and presentation modality within virtual reality games can be crucial factors that can possibly affect players’ interests and actual behavior. In addition, narrative creation using a known universe as opposed to a brand-new one could also affect the players’ interest depending on the popularity and reception of the franchise from the known universe among the target population. A good game narrative should incorporate attractive story elements tailored for the target population and, more importantly, tie organically with the game mechanics and gameplay. The virtual reality environment provides a uniquely immersive environment that could render narratives more engaging and personal than a conventional media device with its presence-related cognitive heuristics (Sundar et al. 2017). Accordingly, specialized virtual reality production may be employed to create a truly immersive virtual reality narrative gaming experience, allowing infinite interaction and complete immersion. Despite these technical limitations, we were still able to observe multiple positive behavioral and psychological results for the narrative element incorporation. Further studies using virtual reality-specific narrative videos should be conducted to explore the potential of immersion and engagement in virtual reality games.

## Conclusion

We are among the first to demonstrate the effect of narrative element incorporation on fully immersive virtual reality games. It also provides multiple valuable practical insights for the future development and incorporation of narratives into active virtual reality games. Despite that the narrative element was a crude and noninteractive animation compiled by the research team instead of fully immersive and interactive narratives, our narrative element incorporation to active virtual reality games still increased the proportion of time spent in moderate-to-vigorous physical activity and reduced the proportion of non-movement among sedentary college students. Active virtual reality games induced on average 13 min of moderate-to-vigorous physical activity in each game session (around 50% of total play duration across the conditions) and induced higher positive affect and PAE than sedentary virtual reality games.

Narrative production and incorporation of different virtual reality game environments are crucial elements that should be explored in future studies to identify the optimal and boundary conditions for both story and virtual reality game genre development. For example, should narratives be adapted differently for active virtual reality versus sedentary virtual reality games? How can researchers separate the effect of narrative versus narrative gameplay in future studies? What are the longitudinal effects of narrative impact on physical activity and sedentary behaviors? These inquiries could be the beginning of unpacking the fundamental mechanism of narrative’s motivational effect on long-term gameplay and health behavior promotion via virtual reality environments.

## Supplementary Information

Below is the link to the electronic supplementary material.

Supplementary file1 (DOCX 14 kb)

## Data Availability

The datasets generated during and/or analyzed during the current study are available from the corresponding author on reasonable request.
