# Supplementary material for: The effect of narrative element incorporation on physical activity and game experience in active and sedentary virtual reality games
Source: Virtual Real. 2023 Jan 31:1–16. Online ahead of print. doi: 10.1007/s10055-023-00754-7 (PMC9888740; doi:10.1007/s10055-023-00754-7)
Supplement: Supplementary file 1 — Supplementary file1 (DOCX 14 kb) [file 10055_2023_754_MOESM1_ESM.docx]

Table S1. General linear model results for Game Engagement Questionnaire (GEQ) measures, PAE, and MS, expressed as mean and standard deviation, for active virtual reality (AVR) vs. sedentary virtual reality (SVR) games, with and without narrative, after Social Desirability adjustment

|  |  | | Narrative Condition | | Non-narrative Condition | | Game effect  (AVR vs. SVR; Within) | | Condition effect  (Narrative vs. Non; Between) | | Interaction (Game x Condition) | |
| --- | --- | --- | --- | --- | --- | --- | --- | --- | --- | --- | --- | --- |
| **GEQ-Competence** | |  | |  | | **F = 6.01** | | F = 0.17 | | **F = 2.92** | |  |
|  | AVR game | 3.2 ± 1.0 | | 3.1 ± 1.1 | | ***p* = 0.02** | | *p* = 0.68 | | ***p* = 0.10** | |  |
|  | SVR game | 2.9 ± 0.8 | | 3.2 ± 0.9 | | **η_p_^2^ = 0.15** | | η_p_^2^ < 0.01 | | **η_p_^2^ = 0.08** | |  |
| **GEQ-Sensory & Imaginative Immersion** | | | |  | | F = 2.80 | | F = 0.15 | | **F = 3.28** | |  |
|  | AVR game | 3.3 ± 0.8 | | 3.0 ± 0.9 | | *p* = 0.10 | | *p* = 0.70 | | ***p* = 0.08** | |  |
|  | SVR game | 2.9 ± 0.9 | | 3.0 ± 1.1 | | η_p_^2^ = 0.08 | | η_p_^2^ = 0.04 | | **η_p_^2^ = 0.09** | |  |
| **GEQ-Flow** | |  | |  | | F = 2.29 | | F = 0.09 | | F = 0.17 | |  |
|  | AVR game | 3.7 ± 0.8 | | 3.6 ± 0.9 | | *p* = 0.14 | | *p* = 0.77 | | *p* = 0.68 | |  |
|  | SVR game | 3.3 ± 0.9 | | 3.2 ± 1.2 | | η_p_^2^ = 0.07 | | η_p_^2^ < 0.01 | | η_p_^2^ < 0.01 | |  |
| **GEQ-Tension** | |  | |  | | F = 0.42 | | F = 0.34 | | F = 2.50 | |  |
|  | AVR game | 1.3 ± 0.5 | | 1.4 ± 0.7 | | *p* = 0.52 | | *p* = 0.56 | | *p* = 0.12 | |  |
|  | SVR game | 1.7 ± 0.8 | | 1.4 ± 0.7 | | η_p_^2^ = 0.01 | | η_p_^2^ = 0.01 | | η_p_^2^ = 0.07 | |  |
| **GEQ-Challenge** | |  | |  | | F = 0.06 | | F = 1.06 | | F < 0.01 | |  |
|  | AVR game | 2.6 ± 0.7 | | 2.8 ± 0.9 | | *p* = 0.81 | | *p* = 0.31 | | *p* = 0.96 | |  |
|  | SVR game | 2.3 ± 0.7 | | 2.5 ± 0.9 | | η_p_^2^ < 0.01 | | η_p_^2^ = 0.03 | | η_p_^2^ < 0.01 | |  |
| **GEQ-Negative Affect** | |  | |  | | F = 0.09 | | F = 0.06 | | F = 0.02 | |  |
|  | AVR game | 1.8 ± 0.8 | | 1.8 ± 0.8 | | *p* = 0.77 | | *p* = 0.81 | | *p* = 0.90 | |  |
|  | SVR game | 1.6 ± 0.6 | | 1.6 ± 0.5 | | η_p_^2^ < 0.01 | | η_p_^2^ < 0.01 | | η_p_^2^ < 0.01 | |  |
| **GEQ-Positive Affect** | |  | |  | | F = 1.19 | | F = 0.05 | | F = 0.17 | |  |
|  | AVR game | 3.8 ± 1.0 | | 3.8 ± 0.9 | | *p* = 0.28 | | *p* = 0.82 | | *p* = 0.69 | |  |
|  | SVR game | 3.2 ± 1.0 | | 3.2 ± 1.1 | | η_p_^2^ = 0.04 | | η_p_^2^ < 0.01 | | η_p_^2^ = 0.05 | |  |
| **PAE** |  |  | |  | | F = 0.01 | | F = 0.07 | | F = 1.04 | |  |
|  | AVR game | 4.3 ± 0.6 | | 4.13 ± 0.7 | | *p* = 0.94 | | *p* = 0.80 | | *p* = 0.32 | |  |
|  | SVR game | 3.7 ± 0.9 | | 3.70 ± 0.7 | | η_p_^2^ < 0.01 | | η_p_^2 <^ 0.01 | | η_p_^2^ = 0.03 | |  |
| **MS** |  |  | |  | | F = 1.48 | | F = 1.69 | | F = 0.63 | |  |
|  | AVR game | 1.5 ± 0.5 | | 2.24 ± 1.9 | | *p* = 0.23 | | *p* = 0.20 | | *p* = 0.43 | |  |
|  | SVR game | 1.5 ± 0.8 | | 1.90 ± 1.6 | | η_p_^2^ = 0.43 | | η_p_^2^ = 0.05 | | η_p_^2^ = 0.02 | |  |

Abbreviations: PAE = Physical Activity Engagement; MS = Motion Sickness.

Significant or borderline effects are bolded.
